# Positive leadership action framework: Simply doing good and doing well

**DOI:** 10.3389/fpsyg.2022.977750

**Published:** 2023-01-04

**Authors:** Dulce M. Redín, Marcel Meyer, Arménio Rego

**Affiliations:** ^1^Department of Business, University of Navarra, Pamplona, Spain; ^2^Católica Porto Business School, Universidade Católica Portuguesa, Portugal; ^3^Business Research Unit, ISCTE-IUL, Instituto Universitário de Lisboa, Porto, Portugal

**Keywords:** positive leadership, virtue ethics, positive organizational scholarship, practical wisdom, organizational virtuousness

## Abstract

This article presents the Positive Leadership Action Framework (PLAF) to structure Positive Leadership (PL). The novelty of the PLAF is that it incorporates the connections of PL to positive outcomes (financial and economic performance and social well-being) and organizational virtuousness. Also, it acknowledges its conditional nature on the virtues to achieve flourishing within the organization and society at large. We argue that the leader’s actions function as the engine for positive change within the organization, bridging the gap between individual virtues and organizational virtuousness and creating a feedback loop among both. To develop a positive organization, a leader needs to create positive assumptions among (and about) coworkers, positively impact the personal and professional development of employees, and balance positive formal and informal conditions at work. To do so, it is a *sine qua non* condition that the positive leader fosters his/her personal development by exercising the virtues and developing practical wisdom. In this way, the positive leader automatically provides followers with a vision of the final end towards the common good and achieves to set his/her organization on a pathway towards excellence.

## Introduction

“Your mind is a powerful thing. When you fill it with positive thoughts, your life will start to change.” This proverb from a recent BBN Times article from Vartika Kashyab (Kashyab, 2019), Marketing Manager at ProofHub and one of the LinkedIn Top Voices in 2018, exemplifies one of Positive Organizational Scholarship’s key messages: You cannot become great (as a leader or an organization) by only eradicating your weaknesses – focusing on the negative. To become truly great, it is also necessary to build upon your strength/talents – concentrating on what is positive.

Positive Leadership (PL), an offshoot from Positive Organizational Scholarship (POS), combines research from positive social sciences with leadership knowledge derived not only from Virtuous Leadership, but also from Authentic Leadership, and Participatory Organizational Leadership and Empowerment (Meyer et al., 2019). Given its pragmatic orientation^1^, the research field leaves an ample margin in the definition of goals to accommodate a diversity of preferences (Meyer et al., 2019). Thus, it does not come as a surprise that PL research focuses on results as diverse as social benefit (Quinn and Thakor, 2014), peace (Spreitzer, 2007), excellence (Dutton and Spreitzer, 2014), justice (Ambrose et al., 2013), positively deviant performance and human flourishing (Cameron, 2008) and human progress (Rego et al., 2012), among others. Still, despite such a vast range of research foci, there seems to be a consensus in the literature (Spreitzer and Cameron, 2012) that, in terms of ends, positive businesses and their leaders do well and do good. Cameron, Quinn and Caldwell (2017, p. 60) explain: “From a POS perspective, there is very little conflict […] between doing well and doing good.” ‘Doing well’ refers to “marked improvements in terms of multilevel performance including economic, human, and environmental aspects, indicating the magnitude of change in an upward trajectory and highlighting future viability and sustainability” (Meyer, 2015, p. S184). ‘Doing good’ implies “undertaking actions to create a beneficial and sustainable situation for a company, the stakeholders and the community, the environment, and for society as a whole” (Meyer, 2015, p. S188). Doing good and doing well are very much interlinked.

Cameron and Spreitzer (2012), by indicating four key attributes of *positive* shed further light on the defining features and boundaries of PL. They indicate that positive, first, refers to a change of perception concerning challenges, adversities, problems, and so forth towards a more favorable point of view. Second, it denotes positive deviant outcomes, meaning that they depart from the norm of reference group in honorable and intentional ways (Spreitzer and Sonenshein, 2003). Third, it represents “an affirmative bias” (Cameron and Spreitzer, 2012, p. 3), which implies a focus on desirable practices, enhanced individual and organizational resources, and the creation of upward spirals in human systems. Fourth, it is connected to the idea of virtuousness, defined as “that which is good in itself and is to be chosen for its own sake” (Cameron and Spreitzer, 2012, p. 3). These four core aspects, derived from POS, make PL unique. They differentiate PL from other so-called positive leadership approaches such as Authentic Leadership, Servant Leadership or Humble Leadership.

The study of virtuousness (also frequently used as a synonym of positive) resulted in the concept of organizational virtuousness which, in turn, became a core concept in positive organizational studies (Cameron, 2003; Cameron and Winn, 2012). Yet, despite virtuousness’ central role, several authors have pointed out that the underpinning of the concept is not yet clearly articulated (Sison and Ferrero, 2015; Meyer, 2018; Newstead et al., 2018). This has been slowing down the advancement of research, including PL. Besides, the connections between individual virtue and organizational virtuousness are not well-defined, leaving the terms to be used interchangeably in scholarly literature. This interchangeability is problematic, for both research and practice, because what happens at the organizational level is not the aggregate of what happens at the individual level. For example, an organization employing many individuals demonstrating individual virtues and strengths is not necessarily a virtuous organization – rather such virtues and strengths may be necessary to deal with a toxic organizational leader. This has added to a poor understanding of the nature of PL and the interrelations among the leader, the organization, and society at large.

Despite these drawbacks, PL has been gaining ground (Lam and Roussin, 2015) and promises to further strengthen the role of virtues in leadership. This is particularly encouraging in the context of spiraling business scandals, global inequalities and the disastrous consequences of a financial and economic crisis that threatens to be taken to new heights with a new recession of the global economy just around the corner. In the words of Ashford and DeRue, “At a time when the status quo is unsustainable and a “new normal” is required, the need for exceptional leadership at all levels of organizations has never been greater” (Ashford and DeRue, 2012, p. 146).

In this paper, we present the Positive Leadership Action Framework (PLAF) to structure PL. Drawing on Aristotelian virtue ethics, we follow Newstead et al. (2018) in interpreting virtue as a mechanism that gives rise to action, which becomes the linchpin between individuals and groups and the enabler of *eudaimonia*, i.e., goodness and happiness in a way that serves the common good (Wright and Goodstein, 2007). Through the PLAF we effectively connect the virtue of the leader with organizational virtuousness and the positive outcomes of the corporate activity (financial and economic performance and social well-being) associated with PL. Besides, we shed light on what exactly positive leaders do. Hence, this article’s contribution is threefold. First, we clarify PL and strengthen the research field’s composition and underpinning. Second, we translate existing conceptualizations of PL into practical guidelines so that exercising managers might find value in applying our framework. Finally, we provide a solid foundation for the reconciliation of virtue ethics approaches to leadership –with a locus on the individual– and those that arose from POS that attempt to measure and develop virtuousness at the organizational level.

The paper proceeds as follows. We provide a literature review to clarify the main pillars of the PLAF: (individual) virtue, organizational virtuousness, and the nature of positive business. Then we define the actions that characterize a positive leader and present results that can be linked to these activities. We discuss how the PLAF might change our perception of doing business and, then, summarize the contribution of the study and present areas of future research.

## The cornerstones of PL

PL is about identifying and building upon human strength (Manz et al., 2008). It is about creating resilience in organizations and fostering human flourishing (Cameron, 2008). Research centers on what leads to prosperity and human excellence, as well as extraordinary individual and organizational functioning. However, to our knowledge there is no theoretical framework of PL that comprises all these features. Especially the link between the individual and the rest of the organization is still underdeveloped.

**Figure 1:**
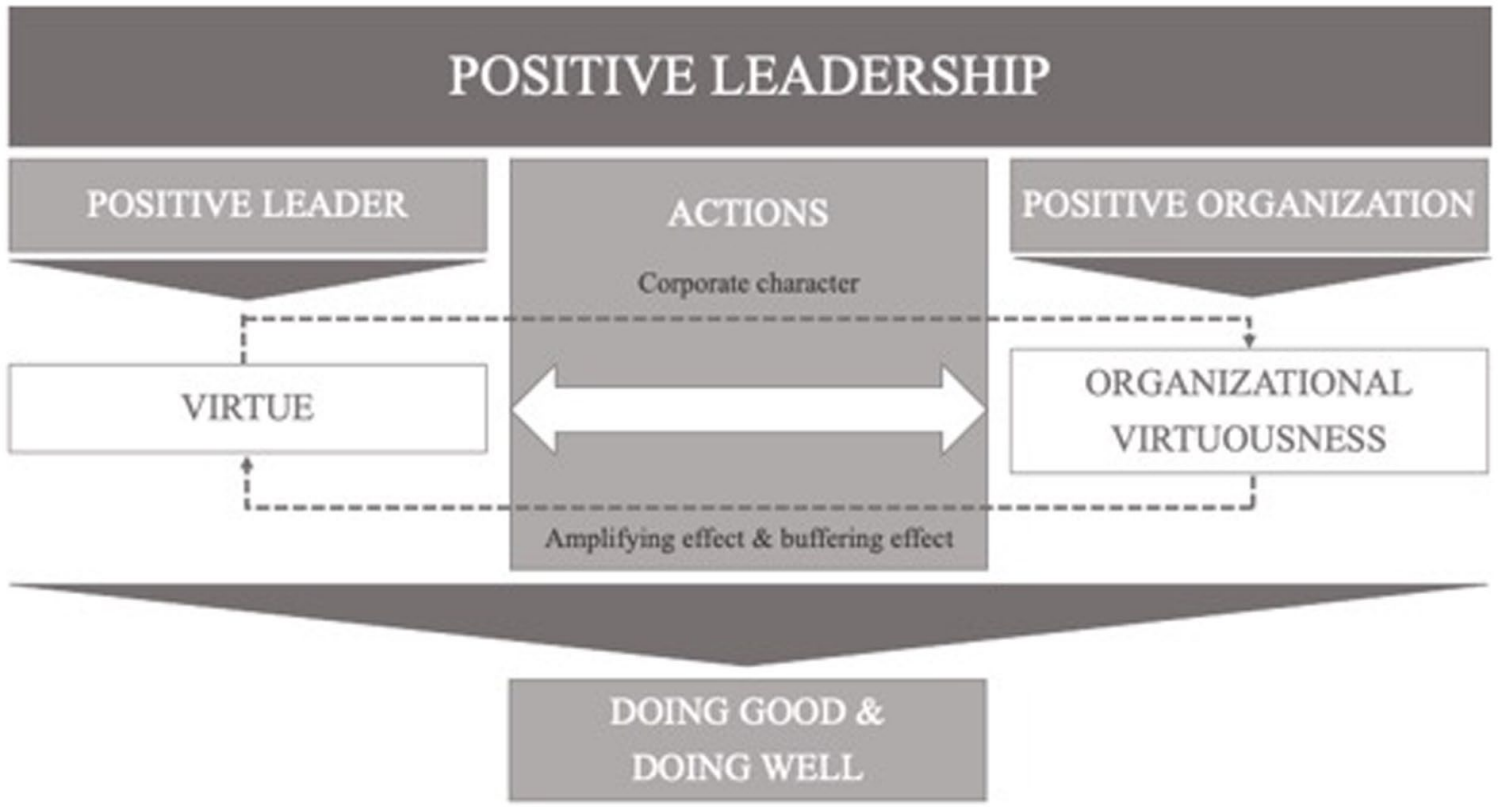
The building blocks of the PLAF.

We claim that the nature of PL should be set upon three interconnected building blocks: The first one is the Aristotelian perspective of the role of virtue in the corporation – an aspect that still needs headway in PL and POS (Meyer, 2018). The second is (organizational) virtuousness, which is defined as a key aspect of POS (Cameron and Winn, 2012). The last block is, then, adopted from positive businesses – the idea that corporate performance is directed towards excellence, always considering the financial or economic point of view as well as the well-being of the individuals and communities involved in its activity (see Figure 1).

### Building block 1: Virtue

Virtue is human excellence that emerges from an inclination, which gives rise to actions that conform habits, and eventually constitute a character, anchored to a specific account of human nature (communal and relational) and its end –*eudaemonic* well-being or meaningful happiness (Sison and Ferrero, 2015). In other words, virtues typically describe character traits considered to be ‘excellent’. These character traits are built usually through the performance of virtuous habits, which are generally the result of a repetition of virtuous actions. In this sense, it becomes clear that virtues, apart from referring to character, also concern actions and habits. Actually actions can be considered the fundamental cornerstone of virtue because an agent’s emotions, thoughts or inclinations obtain moral relevance only through practice. What is more, Aristotle’s Nicomachean Ethics are primarily based on the idea of ‘proper human functioning’ (*ergon*), which translates into “some sort of life of action of the [part of the soul] that has reason” (Aristotle, 1985, Nicomachean Ethics, [NE]: 1098a). Aristotle’s idea of virtue is a theory of ‘action’ – *eudemonia*, his idea of the supreme goal of human flourishing, cannot be achieved purely through fine reasoning, excellent judgements, or deliberating. There cannot be virtue without ‘doing’ the right thing and turning rational activity into physical action. As Sison (2003, p 22) observed, “most knowledge tends to be of the theoretical kind, while virtue is more of a skill or practice that one learns or acquires by doing.” To transfer this idea to the world of business, just imagine someone that has the most fantastic idea for a new business. Each and every aspect is planned down to the tiniest detail. This business idea, however, will never be developed without the ‘entrepreneur’ taking the necessary steps and turning his plans into action. Like in business, for any virtue theory it is crucial to turn thoughts into reality – to be a doer and not a ‘don’ter’. In this sense, virtue is not so much about *attaining* excellence. In fact, Aristotle knew very well that one life might not be enough to perfect all virtues (NE: 1101a). Virtue is much more about the ‘pathway’ it takes to become excellent; it is about each and every little step we take. To learn from mistakes and to reflect upon our own actions and to steer our own development along the right path.

Being actions the first cornerstone of virtue, a second level of Aristotelian virtue ethics, then, are habits which simply are the result of frequent repetitions of voluntary actions (NE: 1103a). Hence, habits are well-founded dispositions to act in a certain way. The development of virtuous habits from voluntary actions presupposes three different levels of freedom: (1) physical freedom, (2) psychological freedom, and (3) moral freedom. While physical freedom simply refers to the capacity for motion or movement, psychological freedom means that an agent’s decisions and actions are the result of his/her independent will. Moral freedom refers to a choice we are making to strive for something superior and nobler than our natural condition (Meyer and Sison, 2020). Thus, it is through our virtuous habits that we perform more good actions in a better way. Besides, habits generally do allow us to develop our skill-set by continuously training and practicing up to the point that our routines become something like a ‘second nature’. Imagine, for example, the manager of a sales department, who wants to improve her hiring techniques by being more honest. In the best of cases her honesty encourages the job applicants to be honest as well – something that would allow the manager to select better between the possible candidates. In any case, her being more honest would also set an example for co-workers. Eventually, our manager would *be seen* not only as more honest, but also *become* a person that is known to be trustworthy. It would not be surprising if the manager from our little example would be considered a positive example in her department. It is known that leaders, as role models, can have a strong impact on their employees through their behaviors (Cameron and Caza, 2002).

Character, then, is the third level in which we find the virtues. In contrast to a specific behavior, it is more demanding to alter one’s character. This is why any virtue theory which is only based on behavior alone would be considered a rather weak idea of virtue. Character depicts a more whole, comprehensive, and defined picture of human beings than any behavioral description ever could; it displays greater permanence than just single actions or habits, and it is thus why Aristotle considers character states the proper locus for virtue (NE: 1106a). Still, character incorporates a person’s different customs, which, additionally, usually are to be in various phases of development. As Sison (2003, p. 108) argues, “as we may recall, character is what results from habit - or from the combination of different habits that a person develops – as its name in Greek suggests” (see also NE: 1103). One person might at the same time be generous and loyal, both being considered virtues by Aristotle, but also impatient, a vice or deficiency of virtue, and stubborn, a vice or excess of a virtue. “Thus, character describes the entirety of a person’s habits plus their degree of development, providing a person with an inimitable touch” (Meyer and Sison, 2020, p. 284). Even though Aristotle’s original list of virtues goes back to the 4^th^ century before Christ, some of the character traits he described (e.g., honesty, caring, or confidence) are still used by leadership experts to describe good leaders (Farrel, 2011).

Character displays a reasonably thorough image of a human being. Still, one could even go one-step further and look at a person’s lifestyle. It would mean to study a person’s feelings, behaviors, and his/her life as a whole. Going back to the example of virtue being a passageway, that virtue represents many different little steps (actions) to flourish on the way to become excellent, we can understand a lifestyle as an all-inclusive choice – a representation of a person’s aim in life which also corresponds to this person’s ideals and moral point of view (Meyer and Sison, 2020). As Sison (2003, p. 118) observes:

**“The ultimate distinguishing principle in character, therefore, is the use that each individual person makes of his own free will in the myriad of situations that life presents to him. And the most complete and lasting testament of a person’s decisions regarding these matters could be found in his lifestyle choice.”**

Lifestyle logically includes both a person’s personal life and his/her career or professional life. While one might have developed certain character traits typically shown at work, this same person might have developed other virtues in his/her personal life at home. While a manager’s husband might describe his better half foremost as caring and loving, the coworkers might describe her primarily as hard working and consistent. Bringing both spheres together would give us a more complete picture of the manager’s lifestyle – a picture that might not be complete if we look only into one aspect of her life.

Like in our example from above, a person may not display exactly the same virtues or vices at work and at home. This is mostly due to the different roles one takes on in life and which all contribute to create a person’s life story (Sison et al., 2018). However, as Aristotle (NE: 1095a) advises us, even though the same person may play different roles in different settings, all his/her actions should be directed towards “*eudaimonia*” – that what all human beings ultimately seek – a flourishing life. Such a life entails “living well and doing well” (NE: 1098a), and it exemplifies virtue or moral excellence *per se*. Yet, albeit most people agree that they are striving for a thriving life, there are manifold interpretations of what exactly such a life means and encompasses. In the best of cases, however, an employee’s goals and moral points of view are aligned with the vision and values of the organization he/she works for (Meyer and Sison, 2020).

#### Virtue and leadership

Although Aristotle recognizes a person’s environment and social interaction as crucial to exercise the virtues (NE: 1095b), his account on virtue is foremost of an individual nature. As described above, to become virtuous depends on one’s own free will. It is contingent on one’s own decisions and actions and on being consistent in practicing the virtues. Leadership, on the other hand, is mostly interpreted as a bi-directional transformative and intrinsically moral relationship between leaders and followers (Burns, 1978). It is a relational and co-created process involving leaders and followers (Uhl-Bien, 2006; Uhl-Bien et al., 2014). Still, to become a good leader one needs to lead oneself first – develop virtues – in a way that others might want to follow. In that sense, we agree with Sison (2003, p. 67) that “leadership consists in nurturing virtuous action in one’s followers by performing virtuous actions oneself; that is, by giving good example.” Consequently, for the positive leader, as well as for any other leader that rejects force or coercion to shape an organization, it must be of primordial importance to develop ethical qualities by acting morally upright apart from technical and conceptual competence (Ciulla, 2003).

Many existing theories of leadership cite virtues as fundamental to good leadership –e.g.: Pearce et al. (2006), Riggio et al. (2010), Cameron (2011), Lang et al. (2012), Hackett and Wang (2012), Fehr et al. (2015). Extant theories of good –virtuous, moral, ethical– leadership stress the resonance between virtue and leadership, although they do not propose any strategy to connect the praised virtues into the daily practices of leaders in organizations (Newstead et al., 2020). This is part of the contribution of the PLAF, which is presented in section 3.

### Building block 2: Organizational virtuousness

Organizational virtuousness is a core construct of POS and, therefore, crucial to understand the dynamics underlying PL. Generally, it is exhibited through virtuous behaviors, processes, and routines at the collective level (Bright et al., 2006; Cameron and Winn, 2012; Cameron and Caza, 2013). While organizational virtuousness is claimed to be based on Aristotelian thinking (Cameron, 2003, 2008), in contrast to Aristotelian virtue ethics, the focal point of positive organizational studies is the collective level of analysis (Bright et al., 2012). Virtuousness refers to organizations in the first place and only secondarily to individuals (Sison and Ferrero, 2015). This also becomes clear by looking at the two different notions of organizational virtuousness presented: virtuousness *in* organizations and virtuousness *through* organizations. The former refers to the behavior of individuals in organizational settings (Cameron, 2003; Bright et al., 2006; Cameron and Caza, 2013). The latter encompasses enablers that stimulate and increase virtuousness in organizations (Cameron, 2003; Bright et al., 2006), or “formal groups in fostering and sustaining eudemonic action” (Cameron and Caza, 2013, p. 678). Hence organizational virtuousness refers to “organizational contexts where virtues (…) are practiced, supported, nourished, disseminated, and perpetuated, both at the individual and collective levels (Rego et al., 2010). It refers to a context, situation, or condition in an organization that is conducive to the virtues (Meyer et al., 2019).

Organizational virtuousness encompasses the following core characteristics: human impact, moral goodness, social betterment, the heliotropic tendency of human beings, the eudaemonic assumption, the inherent value assumption, plus amplifying and buffering qualities (Meyer, 2018). While most of these characteristics are rather straightforward, especially the amplifying and buffering qualities are worth more explaining. According to the amplifying quality of virtuousness, it creates a self-perpetuating tendency or contagion leading to a self-reinforcing upward spiral in the organizations. By observing others’ virtuous behavior individuals sense an urge to act alike. Witnessing excellent or moral conduct inspires people to replicate such behaviors on their own (Cameron, 2003; Cameron and Winn, 2012). This mechanism is triggered by positive emotions that arise while observing others’ prosocial behavior (Meyer, 2018; Meyer and Hühn, 2020). Thus, positive emotions can help cause positive activities in organizations, which, then, produce more feelings that are positive so that a virtuous cycle is fired up. Employees who act prosocial typically possess virtues such caregiving, empathy, and trust. Virtues like these are crucial when transforming a workplace climate and, what is more, permit a better flow of information, enriched interactions, more dynamism, and more efficient resource sharing between an organization’s members (Dutton and Heaphy, 2003). The buffering quality of virtuousness, for its part, implies a protection or defense against dysfunctions, harm, or illness at individual and group levels. It builds resiliency and robustness (Cameron et al., 2004). Through the buffering effect of virtuousness, organizations are, for example, better protected against the deterioration associated with downsizing (Cameron, 2003). In sum, organizational virtuousness produces self-reinforcing upward spirals of uplifting behavior and cushions individuals and organizations against detrimental and devastating occurrences (Meyer, 2018).

The list of virtues that have received attention in positive organizational studies is large. Just a few examples are humility (Owens et al., 2012; Rego et al., 2017), hope (Luthans and Jensen, 2002; Carlsen et al., 2012; Branzei, 2014), and courage (Worline, 2012). Two virtues, however, stand out as the ones that probably are mentioned most in the literature: forgiveness and compassion (Cameron and Caza, 2002; Dutton et al., 2014; Worline et al., 2017; Meyer, 2018). Besides, two sets of virtues are put forward as representative of organizational virtuousness. One set is comprised of: organizational forgiveness, organizational trust, organizational integrity, organizational optimism, and organizational compassion (Cameron et al., 2004). The second set is formed by: hope-optimism, humility, integrity, compassion, virtuous fulfillment, responsibility, and forgiveness (Bright et al., 2006). Both sets of virtues have been developed with the intention to operationalize and measure the construct of organizational virtuousness. Noteworthy is that, again, both lists include the virtues of forgiveness and compassion.

#### Organizational virtuousness and leadership

On the one hand, leaders are especially important for the dissemination of organizational virtuousness as they have the possibility to create an environment conducive to the virtues. Such an environment might include rules and guidelines that determine a certain course of action that supports the exercise of particular virtues. Apart from introducing such rules and guidelines a leader might as well decide to adopt certain processes or structures that aid the performance of virtues (Cameron, 2003). On the other hand, leaders also have the possibility to enhance the virtuousness of organizations through their function as role-models (Meyer and Sison, 2020).

Cameron (2014), for example, points to three leadership practices that are especially effective in enabling organizational virtuousness: expressing gratitude, institutionalizing forgiveness, and facilitating transcendence. These three leadership actions are certainly not what most people would expect from the manager of a profit-driven enterprise that only cares for money and profits. Still, to show gratitude, compassion or forgiveness and to foster transcendence must not be at odds with economic returns. As Morterson and Gardner (2022) argue, “Leaders do not have to choose between compassion and performance.” Let us just imagine a leader that developed the habit of writing thank you notes to employees that performed especially well. How much would that cost? And how would that impact upon the employee? At the very minimum the employee feels recognized and knows that her work is seen, respected and appreciated.

To show forgiveness, on the other hand, might help to heal relationships. To institutionalize forgiveness also means that an organization accepts that mistakes can and do occur. It assures employees that they can take a certain risk in being innovative and try to do things differently. Surely, mistakes can cost money and damage a firm’s reputation, but by being forgiving leaders also teach employees to feel safer when it comes to raising concerns. This can easily help to prevent malpractice or companywide scandals. Just imagine an employee of a medium size bakery mainly producing pastry and selling its products through various local supermarkets. As it happened the employee experienced an extremely negative day with her grandfather in hospital. At work she could not concentrate and finally realized that she did not correctly mix the ingredients of the bakery’s most famous cake. Should she confess the mistake to her shift leader, or should she risk that customers might call in to complain about the bakery’s product in the following days – or, worst case scenario, some of their direct clients, the supermarkets, cancelling their collaboration?

Furthermore, the opportunity to be forgiven also means to have the possibility for professional and personal development. Hence, Spreitzer and Porath (2014, p. 50) recommend that “rather than pull back on empowerment after a mistake, the leader must look for the learning in the experience to garner thriving.”

Regarding a leader facilitating transcendence^2^ (at work), Cameron (2014) highlights outcomes such as employees being more cooperative, creative, or having greater feelings of well-being. A leader can foster transcendence by, for example, setting ‘Everest goals’ that represent an ultimate achievement and/or extraordinary accomplishment. As Cameron (2014) further explains, these goals are meant to be specific, measurable, aligned with the organization’s purpose, realistic and time bound. Furthermore, these goals must serve some kind of extraordinary, honorable, inherently virtuous idea while also representing a clear contribution and creating positive energy. Summarizing, the array of ideas and tools to promote organizational virtuousness is growing and researchers generally stress the instrumental benefit of organizational virtuousness as well as its intrinsic value. On the one hand, Cameron (2014) assures that activating virtuousness in organizations leads to an upsurge in performance at all levels. On the other hand, Bright et al. (2006, p. 252) highlight that, beyond its positive benefits, virtuousness also nurtures specific actions because they simply are ‘the right thing to do’.

### Building block 3: Doing good and doing well

The term ‘positive’ refers to the values and assumptions that lead to the creation of beneficial situations and marked improvements, which put individuals and organizations on an upwards trajectory toward achieving excellent functioning, assuring profitability in addition to sustainability and social well-being (Meyer, 2015). Positive businesses do well and do good. ‘Doing well’ implies high deviant performance and profitability. Corporate activity is evaluated both in terms of financial and economic factors, and human and environmental performance indicators. As Sison and Ferrero (2015, p. S85) stress, there is a *eudaimonic* resonance in the idea of ‘doing well’: ‘doing well’ and ‘living well’ is analogous to ‘being happy’ or living a flourishing life. Happiness is desirable in itself and everything else becomes choice-worthy by reference to it. Happiness represents the definitive form of virtue or moral excellence. ‘Doing good’ describes actions –carried out by leaders, but also employees– aimed at shaping a beneficial situation for and within the company, the community and society as a whole. Actions that generate wholeness, create resources, and promote prosperity and mutual benefit count as ‘doing good’ (Meyer, 2015).

A prime example of a positive organization is Kaiser-Hill, a joint venture subsidiary of ICF Kaiser and CH2M Hill Co., two leading environmental management companies, which transformed the Rocky Flats Nuclear Arsenal (Colorado), that produced plutonium and enriched uranium “triggers” for nuclear weapons into a Front Range Wildlife Refuge (Los Angeles Times, 1995; Jacobs, 2005). The project, which can be considered “the largest, most complex environmental cleanup project in United States history” (The Associated Press, 2005), was not only finished way ahead of schedule and under budget, but during the cleanup, Kaiser-Hill produced some 200 technological innovations (Cameron, 2008; Lavine and Cameron, 2012). What is more, Lavine and Cameron (2012), apart from the positive economic/financial achievements, stress the importance given to the human-related performance outcomes such as an enormous reduction of employee grievances, significant advances in stakeholder communication and collaboration, plus notable developments related to the organizational culture. Further examples of positive businesses are Teledyne Brown Engineering, Best Buy, and Southwest Airlines (Spreitzer et al., 2012). “These firms experienced the positive effects of ‘thriving employees’ through reduced health care costs and absenteeism, increased retention, higher productivity, and greater revenues” (Meyer, 2015, p. S182).

As Robert Quinn (2015) explains, most organizations merely strive for survival and not for flourishing. Besides, organizations are neither a hundred percent negative nor hundred percent positive (Quinn, 2015). As we mentioned earlier, the same is true for human beings and the virtues or vices they develop. Hardly ever would we encounter a person that is completely vicious or someone that has reached to advance all the virtues to their maximum. Still, we all know people we admire for how they *are*; people that we have as role-models, because they spread this positive, contagious positive energy that we are able to recognize. Similarly, we are able to tell if, especially concerning the organization we work for, has more positive characteristics than negative aspects. As Quinn (2015) elucidates, to create a positive corporation one needs to understand the organization not as static, but a living system that requires molding on its way to positivity. Therefore, we can also understand fruitful attempts of creating something novel – a new business – from the ashes of a past corporation (Walsh and Bartunek, 2012), the successfully merger of two culturally distinct companies (Cameron and Plews, 2012) or hybrid organizations (Haigh and Hoffman, 2012) as positive businesses. In this sense, it also becomes clear that the purpose of an organization, and with it, the direction it is steered towards is crucial to determine if a business is positive or negative. Regarding the intention and course positive businesses are set upon, Spreitzer and Cameron (2012, p. 86) indicate that “whereas traditionally positive outcomes such as improving the organization, and achieving goals or profitability are not excluded from consideration, POS has a bias toward life-giving, generative, and ennobling human conditions, regardless of whether they are attached to traditional economic or political benefits.” Meyer (2015, p. S175) goes even one-step further and claims: “Profits are considered vital and necessary, but the final ‘raison d’ être’ of positive states and practices is the overall well-being of the stakeholders.”

## The actions of the positive leader

PL scholars like Cameron (2003, 2008, 2014) highlight Aristotelian antecedents for virtuousness through references to virtue and flourishing. The terms ‘virtues’ and ‘virtuousness’ are used interchangeably in the PL literature, usually combined with concepts and methods –such as quantitative analysis– proper to POS and also positive psychology. Even though both terms denote a human good –i.e., a desirable aspiration for human beings– with some intrinsic goodness –i.e., a perfective state–, there are strong conceptual differences between the positive idea of virtuousness and the Aristotelian notion of virtue, which have been stressed both by researchers from POS on the one hand such as David Bright, Bradley Winn, and Kim Cameron who – among others – led the symposium ‘Virtue, Virtuousness or Vice: Conceptual Tensions in the Study of Virtue in Positive Organizational Scholarship’ at the Academy of Management Annual Meeting in 2011 (this symposium had several subsequent editions) and virtue ethicists such as Alejo Sison, Ignacio Ferrero. These differences are substantial and make it practically impossible to fully merge both concepts. Still, it should be possible to merely join both constructs within the same framework. The key to do so is to combine the emphasis of virtue on the individual with the focus on the collective dimension of organizational virtuousness. Two facts support this idea. First, this is viable because both notions of ‘virtue’ and ‘virtuousness’ acknowledge the relational and social dimension of human beings (Sison and Ferrero, 2015). Second, it is doable considering that virtue is a mechanism that gives rise to action (Newstead et al., 2018), and action can be interpreted as the key connection between individuals and groups and the enabler of *eudaimonia*, i.e., goodness and happiness in a way that serves the common good (Wright and Goodstein, 2007). Hence, our next goal is to determine: *What are the specific actions that positive leaders perform?*

To do so, we run a literature-based and exploratory study to determine the sort of actions that characterize a positive leader, and which constitute the adhesive among virtue, organizational virtuousness and positive business outcomes as described in the PLAF (see Figure 1). Thereby we draw on research from Meyer (2015) who sets out to clarify the meaning of *positive* by analyzing literature that specifically deals with positive business transformations (including numerous real-life examples of positive businesses) and ‘how *leaders* can create more positive systems, careers, and wellbeing in the workplace’ (Spreitzer and Cameron, 2012, p. 88; *italics added*). In his paper, Meyer (2015) gives an exhaustive insight into what is actually *done* in positive businesses – and the results, which can be expected. By way of induction, we continue to pattern and group the data provided by Meyer (2015) on positive leadership activities (i.e., doing good) to shed further light on the notion of positive leadership and facilitate practitioners with a tool that gives explicit advice on how to become a positive leader (see Table 1).

**Table 1:** Positive leadership actions.

POSITIVE LEADERSHIP ACTIONS	
**CREATE POSITIVE ASSUMPTIONS (ABOUT THE FUTURE)**	
Create hope, inspiration, gratitude, and joy Generate interest Give hope Foster optimism Help members to see life-giving possibilities	
**BASIC LEVEL ACTIONS TO FOSTER POSITIVITY AT THE WORKPLACE**	
**Create a positive formal environment**	**Foster positive professional growth**
Share knowledge / information • Making a long-term commitment to employees • Initiate communications / establish open communication channels • Involve employees in decision making • Paying above-market wages that enable a better quality of life • Treat workers fairly and provide safe and healthy working conditions • Offer multiple benefits for employees (i.e., generous health benefits, child care) • Create safe environment for the employees • Offer incentives to reward safety / collective rewards and compensation • Create a climate that promotes diversity • Minimize occurrences of incivility/Create a more civil, positive culture • Enable decision-making discretion, provide information and feedback • Facilitating dialog and conversation • Foster transparency • Inspire relationships	Help with professional activities • Inspire valuable achievement • Give support to people • Mentor employees • Empower employees • Challenge and support people while they engage in new behavior • Awake potential • Facilitate learning, help to realize potential • Include, engage employees • Elevate, align, and magnify strength • Foster learning at work • Raise job satisfaction.
**GO ABOVE AND BEYOND TO ACHIEVE INDIVIDUAL AND ORGANIZATIONAL FLOURISHING**	
**Create a positive informal environment**	**Foster positive personal growth**
Demonstrate compassion • Value people • Encourage flourishing by inspiring positive emotions, creating positive relationships and articulating meaning • Foster trust and collaborative coherence • Create an environment of support and understanding • Create collaboration and built a level of commitment and ownership • Improve employee’s non-work life • Create a sense of global identity – a sense of belonging • Construct greater meaning • Promote climate of respect and trust / create inclusiveness • Built energy and excitement • Foster commitment, honesty, trustworthiness, and credibility • Reduce social pressure at home → improve family life for employees	Motivate to grow • Build up emotional, cognitive, and relational resources • Activate energy • Bring out the best in human systems • Helping their members (sift through the ashes) • Create a positive impact on employees • Avoid burnout and improve health • Enable vitality

Adapted from: Meyer (2015).

The first group is *‘create positive assumptions about the future’*. It contains actions such as creating inspiration, gratitude, and joy, to foster optimism or to generate hope. To create positive assumptions in followers about the future is crucial for developing an organizational culture that fosters flourishing. “[…] a positive lens focuses attention on the life-giving elements or generative processes […]” (Cameron and Spreitzer, 2012, p. 2). Today’s business world is mostly about solving problems, reducing uncertainty, shattering resistance, and beating the competition (Quinn and Wellman, 2012). PL is different. By changing the focus to the positive, it opens up new ways of thinking, perceiving, and understanding. A world of new possibilities emerges. In a certain way it is similar to the Chinese concept of yin and yang; recognizing that two apparently opposite forces may actually be interconnected, interdependent and complementary. To create positive assumptions about the future does not simply mean to convince others to see the famous half-filled glass of water as half full; it signifies instead to create hope, be inspirational, awaken interest and, in general, to nurture optimism. It also includes being thankful and joyful, positive emotions that, for example, can help to foster learning at work (Ashford and DeRue, 2012). Optimism and hope have been shown to positively influence employees’ relationships and their frame of mind and, thus, to improve their individual and interpersonal functioning (Peterson, 2000). Furthermore, Bakker et al. (2008) stress that optimism and positive self-esteem or a positive belief that good outcomes will be experienced are antecedents to employee engagement which, in turn, is related to workplace well-being (Schaufeli et al., 2006). Hope aids employees to build up a positive identity (Carlsen et al., 2012). Snyder et al. (2002) found hope and optimism to be an essential part of empowerment; important for goal achievement and agency. What is more, optimism and hope are linked to performance improvements and more perseverance (Cameron, 2003). Inspiration and interest signify passion. Passion is an essential part of Duckworth’s (2017) concept of ‘grit’. Grit, the combination of passion and perseverance, has been shown to be crucial for success in various disciplines such as sport and business (Duckworth, 2017). Overall, a leader who achieves to create positive assumptions in her organization is likely to support the wellbeing of his/her employees and to lead the organization towards economic success.

The second group is called *‘create a positive formal environment’*. This group takes together actions that a positive leader does to improve the work environment such as having a transparent and engaging communication with employees, establishing a fair and safe working ecosystem for employees, in terms of working conditions, rewards and benefits, and a diverse climate that fosters inclusive relationships. Sharing knowledge and information, involving employees in decision making, facilitating dialog and conversation, and providing feedback contribute to creating psychological safety (Pearsall and Ellis, 2011; Hirak et al., 2012; Newman et al., 2017) which creates an atmosphere that ensures that employees will feel comfortable speaking up with ideas, questions, concerns, or mistakes. When employees feel that their contribution is welcome and criticism is truly constructive, they bring in their best version and they feel co-participants in the company’s objectives and purposes (Van Tuin et al., 2020). PL demands an inclusive approach to recognize and harmonize the unique potential of each individual to pursue the positive goals of the organization (and articulate those within the personal goals of the agents). This is the only way in which the common good can be understood, as the work in common (Sison and Fontrodona, 2012) of all the members of the organizational community. Hence, creating a climate of transparency, diversity and inclusiveness is a responsibility of the positive leader. Beyond that, such a leader treats his/her subordinates and colleagues well and this should be shown through good formal working conditions that frame the labor relationships. Having fair and competitive remuneration packages, benefits and rewards are necessary conditions to show a genuine long-term commitment to employees (Goswami and Urminsky, 2017; Woolley and Fishbach, 2018). In sum, a positive leader that wants to lead the organization towards the common good must create an inclusive atmosphere in which corporate members see their potential contributing to the organizational goal and feel valued for their unique input.

The third group is labeled *‘create a positive informal environment*’. This group contains actions, which a positive leader performs to foster a welcoming, pleasant environment at work. Actions within this group of leadership activities include demonstrating compassion, fostering trust and collaborative coherence, creating collaboration and building up a level of commitment and ownership among employees. It also contains actions that directly relate to the employee’s non-work life such as reducing employees’ social pressure at home. The positive leader fosters a climate of belonging to the organization founded in trust and respect, so that employees feel valued, supported and committed (Cuadrado et al., 2016). She must encourage flourishing in a way in which none is left behind (Sison and Fontrodona, 2012), i.e., flourishing together. It is important to align the working and non-working spheres of life to avoid potential moral stress (MacIntyre, 1999; employees and leaders themselves are also husbands, mothers, and friends) and articulate meaning with coherence in the different spheres of life and the corresponding specific purposes in each of them, subordinated to the final end.

The fourth group of positive leadership activities is called *‘foster positive professional growth*’. It includes deeds such as helping employees with professional activities, inspiring valuable achievement, giving support to people, mentoring and empowering employees, and also challenging and supporting employees while they engage in new behavior. The role of the positive leader goes beyond professional obligations towards subordinates and colleagues, as he/she functions as a model practitioner that inspires employees (Sims and Brinkman, 2002) through his/her exemplarity and mentoring, rather than focusing on control and monitoring. The positive leader helps, gives support, and challenges others to unfold their potential. Job satisfaction is an important element to foster positive actions and outcomes (Meyers et al., 2013).

The final group is termed ‘*foster positive personal growth*’. This group collects actions, which positive leaders do to help their employees to understand and develop themselves in order to achieve their fullest potential. This group includes actions such as motivating employees to grow and to build up emotional, cognitive, and relational resources, creating a positive impact on employees, enabling their vitality, and helping them to avoid burnout –improving their health. Although not explicitly presented in our table, this also implies to consider leisure time an important aspect. “Leading companies have added amenities focusing on work – life balance, relaxation and leisure activities” (Twenge et al., 2010, p. 1118). This fourth group of actions is complementary to the third one, following the idea that professional and personal spheres of life should be ordered with coherence towards the final end (MacIntyre, 1999). If organizational virtuousness is genuine and it is fueled by the individual actions of people that conform the organization, employees should look for positive actions and excellences also in their personal lives, beyond their professional roles and obligations.

**Figure 2:**
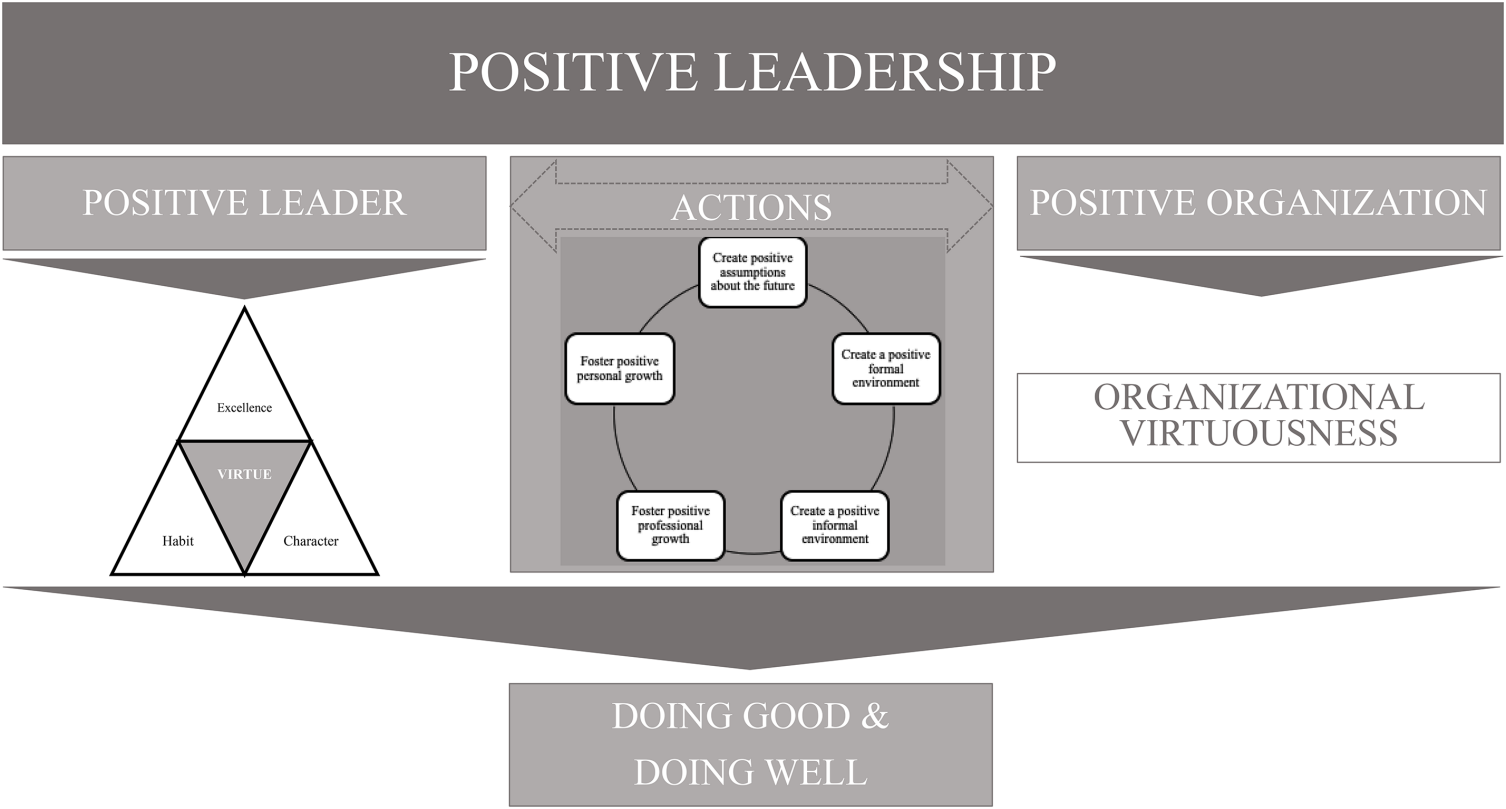
Detailed positive leadership action framework (PLAF).

Together these five groups form the motor of the Positive Leadership Actions Framework (see Figure 2).

## Discussion

Virtue –as understood in Aristotelian virtue ethics– is the basis for organizations to provide the meaningful human connection that members desire (Newstead et al., 2018). Nowadays, our *polis*^3^ is our workplace, since it is where individuals look for meaning, belonging and chances to flourish (Michaelson et al., 2014). Today identities are derived essentially from what we do and where we work, rather than from who we are and where we come from (Newstead et al., 2018). Hence, “the polis is the cradle of virtue” (Hartman, 2013, p. 40). Organizations, understood as communities based on human relationships, provide the context in which individuals look to be meaningful (Solomon, 1993, p. 84). What is more, even though virtue theory typically puts the emphasis on character and individual behavior being a generative mechanism giving rise to virtuous actions (Newstead et al., 2018), still stresses that virtues to be developed and performed also need the collective (Sison and Ferrero, 2015). In this way virtue acknowledges the social or relational dimension of human beings, alongside with an individual one. Hence, to achieve excellence, one must pay attention to both the individual and the social dimensions. It is just this dual facet that enables the extension of virtue from the leader to subordinates and colleagues, and to the organization at large. The extension of virtue to the corporation has been captured by notions such as ‘corporate character’, ‘corporate climate’ or ‘corporate culture’ (*cf.* Moore, 2008, 2015; Bright et al., 2014). In this sense, virtue cannot be understood without the background of the political community and virtuousness without the individual. In a society such as ours, where the *polis* is largely identified with our workplace (Michaelson et al., 2014), it seems reasonable to consider both ‘virtue’ and ‘virtuousness’ as relevant elements for the daily activity of the (positive) organization. Besides, it is exactly this everyday activity, constituted by the countless tiny acts of the organization’s stakeholders, that propels or diminishes the positivity or virtuousness of the organization.

We have presented a variety of activities (see Table 1) to help leaders start practicing positive actions (e.g., to demonstrate compassion, to be honest, to foster learning at work, etc.) which can help them personally to develop virtues and/or foster positive behaviors among their followers. This list of activities is certainly not a closed list. It is merely to be understood as *a representative catalog of actions that are typical in positive businesses* and that research has confirmed to lead to beneficial outcomes for individuals, an entire organization, or even society as a whole. These activities which can be deemed as ‘doing good’ (in POS) are mostly easy to integrate into businesses’ daily routines (e.g., inspire relationships). However, we are not arguing here that a leader needs to integrate all these activities at once. We are simply offering a possibility to start putting a further organization on its pathway to positivity as the way to positivity or excellence is a long one that cannot be walked in 1 day alone.

Furthermore, while patterning and grouping these activities we created five clusters of positive actions. On the one hand, we distinguish between creating a positive *formal* and *informal* environment. On the other hand, we also set fostering positive *professional* apart from *personal* growth. These four groups are foregone by the first set of actions ‘to create positive assumptions (about the future). The fact that we differentiate between formal and informal and between professional and personal, however, does not mean we want to eschew one from the other. On the contrary, we understand that in both cases one cannot exist without the other. While the personal development of a person is much interwoven with its professional growth, it seems impossible to reduce a work environment merely to its formal aspects; this would simply go against human nature and the social dimension of excellence. What is more, “there is significant conceptual confusion in this research area, notably between formal and informal practices, or organizational and managerial practices” (Aubouin-Bonaventure et al. 2021, p. 2). Concerning our framework, therefore, we additionally differentiate between (a) basic level actions to foster positivity at the workplace and (b) actions that go above and beyond to achieve individual and organizational flourishing. While we deem activities to improve the formal environment and foster the professional growth of employees to be deeds that help employees excel and thrive mostly at their job, activities regarding the informal environment and the personal growth of employees directly aim at the human being as a whole to create personal and organizational flourishing.^4^ Thus, this paper argues that in organizations the borderlines between formal and informal and between personal and professional must be permeable and flexible, and that all these spheres must be ordered and aligned with coherence towards the final end. Such as expensive, formal, high-potential talent programs for selected individuals only is an antiquated concept, it is somewhat unrealistic that we can separate, for example, the formal from the informal work environment with a clear-cut line – especially in times of crisis when workers are, for example, asked to switch from working on site to the home office in 1 day. In this sense, the point of view implicit in positive leadership to separate formal supervision from leadership opens a whole lot of new ways of thinking about leadership. To balance the different positive activities and, especially, to manage the interplay between activities related to the creation of positive formal and informal work environments and the actions linked to fostering the employee’s personal and professional growth it is key that a leader develops the virtue of *phronesis* (practical wisdom); a virtue mostly neglected until now in POS (Meyer, 2018). In the Nicomachean Ethics, practical wisdom (phronesis) is the virtue of choosing the suitable means to the right end (NE: 1144a). It means doing the right thing, the right way, for the right purpose, and in the right circumstances (NE: 1126b); performing the morally right action correctly. As Aristotle lets us know, one way to comprehend practical wisdom is that the virtues (e.g., care) mark the right target and practical wisdom, then, gives us the ability to reflect correctly about the means to achieve the target (Aristotle, 1991: 1144a). In this sense, wisdom rides herd on other virtues, enabling people to resolve conflicts among virtues, to find the ‘mean’, and to tailor behavior to the demands of the specific situations they face (Schwarz, 2011). A person that has the virtue of practical wisdom is typically described as someone that has a marked, ample understanding of the world. It is someone who knows what to do in unlikely and uncertain circumstances. Hence, practical wisdom enables the leader to develop a correct view on particular, contingent issues (Meyer et al., 2019). It is a “kind of knowledge of how to act in situations that cannot be judged by applying algorithms (rules of action), but only by thoroughly understanding the concrete situation at hand and judging what to aim for in this particular case” (Svenaeus, 2017, p. 294). For example, practical wisdom enables a leader to know if an employee is ready to accept more responsibility in terms of more decision-making power at work, or how she can best assist a member of staff to find equilibrium regarding her work-life-balance. Not every employee might be open to efforts of creating transparency or collaboration with other departments. Other employees might be rather reserved when it comes to their private life. Therefore, practical wisdom is crucial as it enables leaders to see and achieve what is good for all (Moskop, 1996). Instead of just following guidelines, instructions, or even deontological norms, a leader that has practical wisdom rather relies on a profound reflection-action process to do the right thing (Meyer and Rego, 2020). In sum, practical wisdom is at the core of virtue ethics and it is a key characteristic for leaders. It is certainly a crucial requirement to start developing *phronesis* for each and every person that embarks on the path of positive leadership. As Fitzgerald (2021) points out, nowadays it is key for organizations to find CEOs that do listen to others, have a high level of empathy, are able to persuade the organization’s stakeholders to focus on a common goal, and to communicate clearly.

A further aspect that comes to light when looking at leadership through the positive lens is that it is much more than simply a superior rank in a hierarchy, having the biggest paycheck, or the grandest office on the top floor. Surely, such as Ashford and DeRue (2012) confirm, most of the leadership research after the start of the new millennium associated leaders with formal supervisors, however, positive leadership can be practiced on each and every corporate level without holding any official powers over other staff members. “In a time of rapid technological change and economic uncertainty, businesses that thrive will do so through empowering employees at all levels to take an active role in leading themselves and their organizations to success” (Arnander, 2012, p. ix). What is more, being a positive leader means to have a positive attitude towards life *and* to have others’ wellbeing at heart; to strive towards the *common good*. To find meaning in one’s work, to motivate others to grow, to be honest, to promote a climate of respect and inclusiveness, or to help co-workers through difficult times does not require any official job title. Besides, one cannot be forced to be a positive leader. Leadership comes from within, it is about choice and actions based on these choices.

Mark Sanborn highlights three important points about how everybody in an organization can make a difference by taking on the role of a ‘leader’ (Duncan, 2018). First, he stresses that leadership should be the result of a wish to contribute. Second, it is crucial to take initiative. Third and finally, one must be willing to go above and beyond the call of duty. Aristotle would have agreed with these three points and, in this sense, such as Donald McGannon once powerfully declared: “leadership is not a position or title, it is action and example.”

## Limitations and future research

The main limitation of our study is the somewhat restricted nature of the literature upon which we focused the exploratory study of the actions of the positive leader, even though we consider it representative. Future research should extend this analysis to an exhaustive review of the literature. In addition, researchers should develop empirical studies focused on specific segments of the PLAF. Promising ideas and research results come from Geue (2018) and Aubouin-Bonnaventure et al. (2021) regarding quantitative studies of positive practices. Finally, we consider that it is necessary to explore further the true nature of organizational virtuousness and its dynamics, in order to be able to develop a more accurate version of the PLAF.

## Conclusion

We have presented the PLAF to explain the nature and dynamics of PL. We claim that PL should be framed at the intersection of POS and business ethics. The concepts of virtue and organizational virtuousness, as well as the model of positive business are the main three pillars of the PLAF. As we have exposed, the PLAF rests on the idea that the leader’s actions function as the engine for positive change within the organization, and they bridge the gap between individual virtues and organizational virtuousness, creating self-reinforcing feedback effects among both. On the one hand, the leader’s virtue is extended to the corporation giving rise to notions such as ‘corporate character’, ‘corporate climate’ or ‘corporate culture’ (cf. Moore, 2008, 2015). On the other hand, organizational virtuousness generates an ‘amplifying effect’ and a ‘buffering effect’. The former creates a positive self-reinforcing tendency or contagion in the corporation, the later acts as a protection against organizational harm or dysfunctions (Cameron and Caza, 2002, 2013; Cameron, 2003; Cameron et al., 2004; Caza et al., 2004; Bright et al., 2006). We also run a literature based and exploratory study to determine the specific sort of actions that characterizes a positive leader. By way of induction and admitting the limitations of our sample –which is certainly not exhaustive, although it is representative– we conclude that in order to develop a positive organization, a positive leader needs to create positive assumptions among coworkers, create positive formal and informal environments, and foster positive personal and professional growth of employees. Our framework of positive leadership has several advantages. First, our framework highlights that leadership must not be complicated and that it can be learned. The actions we propose to create positive businesses are not much more than being a decent human being that has learned to unlock her positive personality being on the lookout to train her virtues. Hence, leadership is nothing that is strictly linked to a corporate agenda purely following the call of only financial goals, but it simply is human development over time. Second, our framework subscribes to the fact that leadership can happen at all levels of an organization. Many of the actions we propose here do not require any formal power whatsoever. Third, our framework can easily be used as a tool to foster PL at work, because we not only present the necessary theoretical explanations, but it can also be applied and tested in real-life situations. It thus allows the learning leader to make valuable experiences that will help her to further develop her skills as a positive leader.

## Data availability statement

The original contributions presented in the study are included in the article/supplementary material, further inquiries can be directed to the corresponding author.

## Author contributions

DR and MM have contributed equally to this work, share first authorship, and contributed to conception and design of the article. DR wrote the first draft of the manuscript. MM and AR wrote subsequent versions of the paper. DR, MM, and AR wrote sections of the manuscript. All authors contributed to the article and approved the submitted version.

## Funding

Research Project PID 2021-124151NA-I00 funded by MCIN/AEI/10.13039/501100011033/ and by ERDF A way of making Europe.

## Conflict of interest

The authors declare that the research was conducted in the absence of any commercial or financial relationships that could be construed as a potential conflict of interest.

## Publisher’s note

All claims expressed in this article are solely those of the authors and do not necessarily represent those of their affiliated organizations, or those of the publisher, the editors and the reviewers. Any product that may be evaluated in this article, or claim that may be made by its manufacturer, is not guaranteed or endorsed by the publisher.

## Footnotes

^1^The pragmatic orientation, visible through the various management tools that POS scholars offer (e.g., ‘Strategies for extraordinary Performance’ or the ‘Fundamental State of Leadership’), differentiates PL from neo-Aristotelian leadership with its purely normative approach.

^2^"Transcendence refers to positive deviance, a sense of profound purpose, and the realization of an ideal" (Cameron, 2014, p. 85).

^3^In the first book of the *Politics* (*Politiká*), Aristotle differentiates between the city (*polis*) and other types of communities or associations such as the family. *Polis*, apart from the more literal translation of city, or city-state, makes also reference to what Aristotle considered and termed a political community (*koinonia politike*).

^4^An example how a company can manage the threshold between formal and informal is Southwest Airlines with its humanistic approach towards people. As Herb Kelleher (2012), founder and past long-term CEO of the airline, explained, Southwest Airlines values its staff not just as workers, but as individuals and pays them enormous amounts of attention. "We grieve with them when they have some unfortunate event in their personal lives. We rejoice with them when they have something in the personal life that is a source of joy. And we try to make our people aware of that we value them as people not just as workers when they are at work" (Herb Kelleher, 2012: 204 s – 237 s).
